# The complete chloroplast genome sequence of *Oberonia seidenfadenii* (Orchidaceae), a rare plant species endemic to China

**DOI:** 10.1080/23802359.2019.1674209

**Published:** 2019-10-04

**Authors:** Ming Jiang, Junfeng Wang, Minghui Chen

**Affiliations:** a Zhejiang Provincial Key Laboratory of Plant Evolutionary and Conservation, College of Life Science, Taizhou University, Jiaojiang, Zhejiang, China;; b Lishui Institute of Forestry, Lishui, Zhejiang, China

**Keywords:** *Oberonia seidenfadenii*, chloroplast genome, phylogenetic analysis

## Abstract

*Oberonia seidenfadenii* is a rare and newly recorded plant species in Zhejiang province, China. In our present study, the complete chloroplast (cp) genome sequence of *O. seidenfadenii* was assembled by using high-throughput Illumina sequencing data. The plastome is 143,062 bp in size, which contains a typical quadripartite structure with a pair of inverted repeats (IR) regions (24,278 bp) separated by a small single-copy (SSC) region (10,224 bp) and a large single-copy (LSC) region (84,282 bp). The cp genome sequence contains 127 genes, including 74 protein-coding genes, 38 tRNA genes, 8 rRNA genes, and 7 pseudogenes. Phylogenetic analysis results indicated *O. seidenfadenii* is a sister of *Oberonia japonica*, with a support rate of 100%.

The genus *Oberonia* belongs to the family Orchidaceae and consists of 150–200 species, and the *Oberonia* plants are epiphytic or lithophytic (Wu et al. 2009). *Oberonia* is a taxonomically complex genus and some species only shows slight differences in the morphology of leaves or flowers (Li et al. 2016). There are about 33 species distributed in China, and 11 of them are characterized as endemics (Wu et al. 2009). *Oberonia seidenfadenii* is a tiny Orchidaceae plant species with distichous-equitant leaves, densely clustered inflorescences, and greenish flowers. *Oberonia seidenfadenii* is a species native to China, and it mainly distributes in Taiwan, Guangxi, Guangdong, and Zhejiang (Huang 2000; Tian et al. 2013). In Zhejiang province, its populations are extremely small, and they are found only in some counties of Taizhou and Ningbo with less than 1000 individual plants. The information on chloroplast (cp) genome sequences of *Oberonia* is very limited, and the complete cp genome of *O. seidenfadenii* has not been characterized. In our present study, we assembled the cp genome of *O. seidenfadenii* by using high-throughput sequencing data, and a phylogenetic tree was generated to reveal its relationship with other species.

Leaf samples were collected at an altitude of 36 m on Toumen Island (28°41′35′′N, 121°46′14′′E), Linhai County, Taizhou, Zhejiang province, China. A voucher specimen (CHS2017009) is deposited at the Molecular Biology Laboratory in Taizhou University. Total genomic DNA was extracted by using the CTAB method (Doyle and Doyle 1987), and a DNA library was constructed. The library was then sequenced on the Illumina Hiseq X Ten platform (Illumina, San Diego, CA). A total of 5.5 Gb raw 150 bp paired-end reads were generated, and the filtered reads were *de novo* assembled by the programme NOVOPlasty (Dierckxsens et al. 2017). The cp genome was annotated by Dual Organellar GenoMe Annotator (DOGMA), tRNAscan-SE, and ARAGORN (Lohse et al. 2004; Laslett and Canback 2004; Wyman et al. 2004; Lowe and Eddy 1997). The plastome of *O. seidenfadenii* (GenBank accession: MN414241) is 143,062 bp in size with an overall GC content of 37.1%. The cp genome consists of two inverted repeat (IR) regions, a large single-copy (LSC) region, and a small single-copy (SSC) region, and the sizes of IR, SSC, and LSC were 24,278, 10,224, and 84,282 bp, respectively. The GC contents of *O. seidenfadenii* IR, LSC, and SSC are 43.7, 34.4, and 27.9%, respectively.

The genome encodes 127 genes, including 74 protein-coding genes, 38 tRNA genes, 8 rRNA genes, and 7 pseudogenes. Among these genes, four rRNAs (*rrn4.5*, *rrn5*, *rrn16*, and *rrn23*), eight tRNAs (*trnA-UGC*, *trnH-GUG*, *trnI-CAU*, *trnI-GAU*, *trnL-CAA*, *trnN-GUU*, *trnR-ACG*, and *trnV-GAC*), nine protein-coding genes (*ndhB*, *rpl2*, *rpl22*, *rpl23*, *rps7*, *rps12*, *rps19*, *ycf1*, and *ycf2*) contain two copies. One copy each of *ndhJ*, *ycf1*, *ndhD*, *ndhF*, *rpl22*, and two copies of *ndhB* genes were identified as pseudogenes.

To understand the phylogenetic relationship with other Orchidaceae species, whole-genome sequences of 26 plants were obtained from NCBI, these included five *Dendrobium* species (*Dendrobium officinale*, *Dendrobium hercoglossum*, *Dendrobium chrysotoxum*, *Dendrobium aphyllum*, and *Dendrobium aduncum*), three *Holcoglossum* species (*Holcoglossum weixiense*, *Holcoglossum nagalandense*, and *Holcoglossum amesianum*), two *Neofinetia* species (*Neofinetia falcata* and *Neofinetia richardsiana*), as well as other 15 species from genera of *Phalaenopsis*, *Vanda*, *Pelatantheria*, *Masdevallia*, *Gastrochilus*, *Epipactis*, *Oberonia*, and *Cephalanthera*. *Burmannia disticha* (Burmanniaceae) was used as an outgroup. A phylogenetic tree was constructed by the maximum-likelihood method using PhyML 3.1 (Guindon et al. 2010). The results revealed that *O. seidenfadenii* grouped with *Oberonia japonica*, a morphologically similar plant species, exhibiting bootstrap support of 100% (Figure 1).

**Figure 1.**
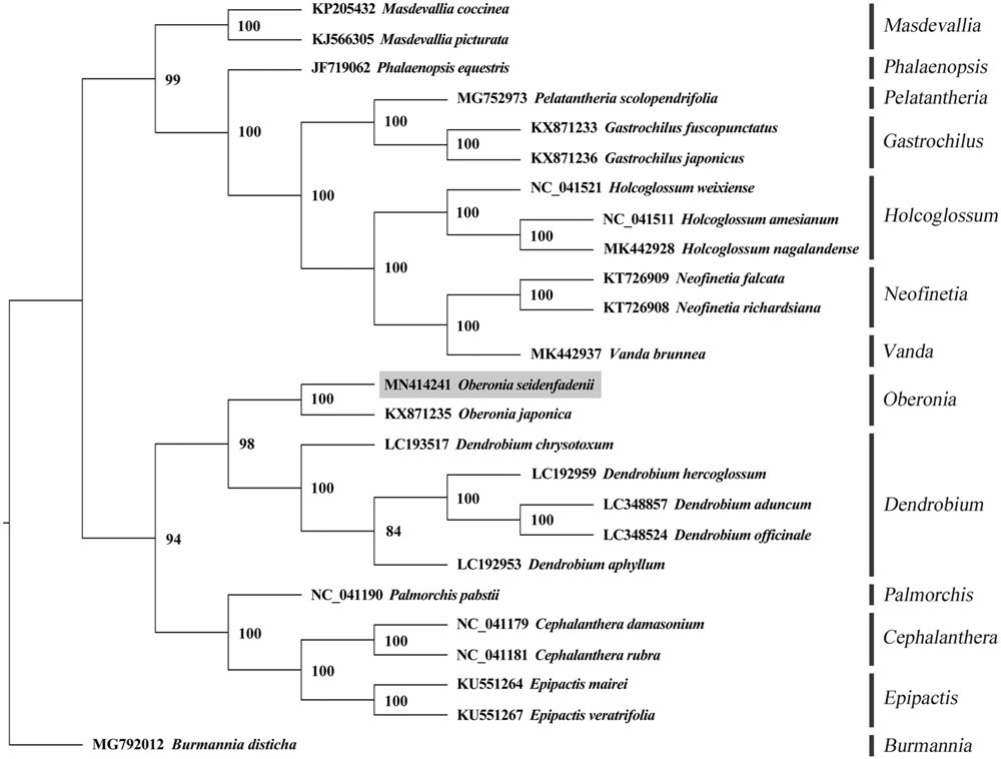
A maximum-likelihood tree based on the complete chloroplast (cp) genome sequences of *Oberonia seidenfadenii* and other 25 Orchidaceae species, with *Burmannia disticha* as the outgroup. The numbers next to nodes are bootstrap support values.
